# 

**Published:** 1905-03-15

**Authors:** 


					﻿Vol. LIX.	MARCH 15, 1905.	No. 3.
THE
DENTAL REGISTER
EDITED BY	,
NELVILLE S. HOFF, D.D.S.\& ,
ANN ARBOR, MICH.
PRICE, ONE DOLLAR A YEAR IN ADVANCE.
Published Monthly by
SAMUEL A. CROCKER & CO.,
Ohio Dental and Surgical Depot,
35 to 39 W. 5th St., Cincinnati, 0.
Entered at the Postoffice at Cincinnati, O., as second-class mail matter.
				

